# Dietary Polyphenol Intake and Depression: Results from the Mediterranean Healthy Eating, Lifestyle and Aging (MEAL) Study

**DOI:** 10.3390/molecules23050999

**Published:** 2018-04-24

**Authors:** Justyna Godos, Sabrina Castellano, Sumantra Ray, Giuseppe Grosso, Fabio Galvano

**Affiliations:** 1 Department of Biomedical and Biotechnological Sciences, University of Catania, 95123 Catania, Italy; justyna.godos@student.uj.edu.pl; 2 Department of Educational Sciences, University of Catania, 95131 Catania, Italy; sabrinacastellano@hotmail.it; 3 NNEdPro Global Centre for Nutrition and Health St John’s Innovation Centre, Cambridge CB4 0WS, UK; sumantra.ray@mrc-ewl.cam.ac.uk (S.R.); giuseppe.grosso@studium.unict.it (G.G.); 4 Integrated Cancer Registry of Catania-Messina-Siracusa-Enna, Azienda Ospedaliero-Universitaria Policlinico-Vittorio Emanuele, 95123 Catania, Italy

**Keywords:** depression, polyphenol, flavonoids, flavanones, anthocyanins, Mediterranean diet, cohort

## Abstract

*Background*: The epidemiological evidence for a relation between dietary polyphenol intake and depression is limited. Therefore, the aim of this study was to assess the association between habitual dietary intake of total polyphenols, their classes, subclasses and individual compounds and depressive symptoms among the participants of the Mediterranean healthy Eating, Lifestyle and Aging (MEAL) study. *Methods*: Demographic and dietary characteristics of 1572 adults living in southern Italy were analyzed. Food frequency questionnaires and Phenol-Explorer were used to calculate habitual dietary intakes of polyphenols. The Center for Epidemiologic Studies Depression Scale (CES-D-10) was used as screening tool for depressive symptoms. Multivariate logistic regression analyses were used to test associations and were expressed as odds ratio (OR) and 95% confidence intervals (CI). *Results*: A total of 509 individuals reported having depressive symptoms. Based on multivariate logistic regression analyses, total polyphenol intake was not associated with depressive symptoms. After adjustment for potential confounding factors, dietary intake of phenolic acid (OR = 0.64, 95% CI: 0.44, 0.93), flavanones (OR = 0.54, 95% CI: 0.32, 0.91), and anthocyanins (OR = 0.61, 95% CI: 0.42, 0.89) showed significant inverse association with depressive symptoms, when comparing the highest with the lowest quartile. Moreover, flavanones and anthocyanins, were associated with depressive symptoms in a dose-response manner. Among individual compounds, inverse association was observed for quercetin (OR = 0.53, 95% CI: 0.32, 0.86) and naringenin (OR = 0.51, 95% CI: 0.30, 0.85), for the highest versus lowest quartile of intake. When taking into consideration the major sources of the polyphenols, only citrus fruits and wine consumption was inversely associated with depressive symptoms (Q4 vs. Q1: OR= 0.51, 95% CI: 0.35, 0.75; Q4 vs. Q1: OR = 0.53, 95% CI: 0.38, 0.74, respectively). *Conclusions*: Higher dietary intake of flavonoid may be inversely associated with depressive symptoms. Further studies are needed to definitively confirm these observed associations.

## 1. Introduction

Depression is one of the major causes of disability worldwide and an important contributor to the global burden of disease [1]. It has been estimated that direct medical costs of managing depression in Europe account for approximately 38 billion Euros [2]. Moreover, leading causes of global age-specific years lived with disability in 2015 showed that, beside para-physiological conditions such as back and neck pain and sense organ diseases, depression had the highest rate of change (making it the third-leading cause of disability worldwide) from 1990 among adults older than 25 years [1]. Therefore, identification of modifiable risk factors and development of preventive strategies are needed in order to decrease the overall burden of the disease.

From a patho-physiological point of view, depressive disorders have been associated with a subclinical inflammatory status characterized by an increase in pro-inflammatory cytokines and neuronal damage (i.e., loss of plasticity) [3]. An intriguing hypothesis proposed over the last years suggests that dietary factors might influence the risk of depression due to various mechanisms, including: (i) antioxidant intake, (ii) increase in homocysteine concentrations and reduced availability of *S*-adenosylmethionine through folate intake, (iii) long-chain omega-3 polyunsaturated fatty acids intake, (iv) influence on monoamine concentrations [4]. There is evidence that diet quality might influence occurrence of depression [5]: dietary patterns characterized by high content of fruit, vegetables, whole grain, fish, olive oil, low-fat dairy and antioxidants and low content of animal foods have been associated with a decreased risk of depression [6]. The recent epidemiological evidence indicates that dietary intake of some plant-based foods and beverages, such as coffee, tea, fruit and vegetable, may be associated with lower likelihood of having depression [7,8]. The common characteristic of the aforementioned food groups is their natural richness in polyphenols, a wide range of compounds with antioxidant and anti-inflammatory properties. Dietary polyphenols have been associated with a number of health outcomes, including decreased risk of mortality, cardiovascular diseases, and several types of cancer [9,10,11,12]. Furthermore, the dietary intake of various classes of polyphenols has been associated with higher adherence to healthy dietary patterns [13], including the Mediterranean diet [14]. Interestingly, several studies conducted in a Mediterranean area showed the association between higher adherence to the Mediterranean pattern with an overall better metabolic health (including dyslipidemia [15], obesity [16,17,18,19,20] and hypertension [21,22,23]), mental-wellness [24], and importantly, psychological resilience [25] and depressive symptoms [26].

The evidence from in vitro and in vivo studies suggests that different dietary polyphenols, such as flavonoids, phenolic acids, lignans, and others, may play a role in brain health, with special regard to depression-linked aspects [27]. Dietary polyphenols have been shown to exert numerous mechanisms that may be involved in depression pathophysiology, including exerting anti-neuroinflammatory properties, suppressing neuronal apoptosis and stimulating adult neurogenesis [28].

Nevertheless, despite promising, the evidence from epidemiological studies is limited. Up to date, solely one investigation on dietary flavonoid intake and risk of depressive symptoms has been conducted [29], while no evidence on other polyphenol classes or individual compounds have been reported. Therefore, the aim of this study was to assess the association between estimated habitual dietary intakes of total polyphenols, their classes, subclasses and individual compounds and depressive symptoms among the participants of the Mediterranean healthy Eating, Lifestyle and Aging (MEAL) study.

## 2. Results

The sample comprised 1572 individuals of mean age of 46.6 years (range 18–92 years). A sensitivity analysis comparing total polyphenol intake between individuals included in the analyses due to availability of the CES-D-10 score and excluded ones revealed no significant differences (629.0 mg/d vs. 671.7 mg/d respectively, *p* = 0.227). Baseline characteristics of the sample are presented in Table 1.

Participants with higher total polyphenol intakes had moderate levels of physical activity, and were moderate or regular alcohol drinkers (Table 1). The individuals in the highest quartile of total polyphenol intake had lower prevalence of hypertension, as well as higher total calorie intake compared to those in the lowest category (Table 1). A higher prevalence of diabetic was found among individuals in the intermediary quartiles of polyphenol intake compared to the extreme ones (Table 1). No other relations with background and lifestyle characteristics were found. Additionally, baseline characteristics of study participants by presence of depressive symptoms are presented in Table S1.

Among the main classes of polyphenols, phenolic acids were the most consumed, despite the widest range of intake (Table S2). Flavanols (including catechins) and isoflavones contributed the most and the least to total flavonoid intake, respectively. Among individual compounds, hesperetin was the most consumed. Regarding major food sources of polyphenols, not all food groups followed a linear trend over total polyphenol intake: indeed, the highest intake of coffee and olive oil occurred in the third quartile of total polyphenol intake, while mean intake of the other food groups followed a linear trend over total polyphenol intake quartiles (Table S2).

A total of 509 (32.4%) cases of depressive symptoms were found in the cohort. No association was found between total polyphenol intake and depressive symptoms (Table 2).

After adjusting for potential confounding factors, none of the polyphenol classes showed linear association with depressive symptoms, despite individuals in the fourth quartile of phenolic acid (OR = 0.64, 95% CI: 0.44, 0.93), and individuals in the third quartile of flavonoid intake (OR = 0.49, 95% CI: 0.32, 0.75), were less likely to have depressive symptoms than those in the lowest category of exposure (Table 2). Among subclasses of flavonoids, a significant contribution to the association of the overall class was provided by anthocyanins (Q4 vs. Q1, OR = 0.61, 95% CI: 0.42, 0.89; *p* for trend = 0.001), flavanones (Q4 vs. Q1, OR = 0.54, 95% CI: 0.32, 0.91; *p* for trend <0.001) and, in a non-linear manner, flavonols intake (Q3 vs. Q1, OR = 0.51, 95% CI: 0.34, 0.77; *p* for trend = 0.034) (Table 3). Among phenolic acids, hydroxycinnamic acids intake was associated with lower odds of having depressive symptoms, despite not in a linear manner (Q3 vs. Q1, OR = 0.65, 95% CI: 0.44, 0.97; *p* for trend = 0.007) (Table 3).

When taking into consideration individual compounds, inverse association was observed for quercetin (OR = 0.53, 95% CI: 0.32, 0.86) and naringenin (OR = 0.51, 95% CI: 0.30, 0.85), for the highest versus lowest quartile of intake. Naringenin was associated with depressive symptoms in a dose-response fashion (Table 4). In contrast, certain compounds, such as hesperetin (OR = 0.67, 95% CI: 0.45, 0.99), kaempferol (OR = 0.63, 95% CI: 0.41, 0.97), matairesinol (OR = 0.32, 95% CI: 0.21, 0.49) and lariciresinol (OR = 0.51, 95% CI: 0.34, 0.76) showed a significant association with depressive symptoms in the third quartile of exposure.

Regarding major food sources of polyphenols, none of the food groups examined showed a significant association with depression, with exception of red wine and citrus fruits; individuals in the highest quantile of intake had lower likelihood of depression than those in the lowest category of exposure (Table 5).

## 3. Discussion

The present study investigated the association between estimated habitual dietary polyphenol intakes and depressive symptoms in a cohort of adults living in the Mediterranean area. Based on multivariate logistic regression analyses, total polyphenol intake was not associated with depressive symptoms. However, several significant associations have been observed for certain polyphenol classes, specifically for high total flavonoid and phenolic acids intake. Among flavonoid class, flavanones and anthocyanin were inversely associated with depressive symptoms, in a dose-response manner. When taking into consideration individual compounds, an inverse association was observed for naringenin and quercetin, for the highest versus lowest quartile of intake. To the best of our knowledge, this is the first study investigating such a broad range of polyphenol classes, subclasses and individual compounds, together with major food sources of polyphenols in order to identify key dietary components possibly implicated in prevention of depressive disorders.

In this study, we found a relatively high prevalence of depressive symptoms (about one third of the sample). It is noteworthy that the tool used to investigate depressive status is able to capture symptoms of depression but should not be considered as equal as diagnosis of depression (which should be performed in the clinical setting). In fact, compared with other studies using this tool, we reported similar prevalence rates [30]. The only epidemiological study on dietary polyphenols and depressive symptoms was focused on flavonoid consumption. The study, conducted on the Nurses’ Health Study and Nurses’ Health Study II cohorts, showed a significant decreased risk of depressive symptoms among women in the highest quintile of flavonol, flavone, and flavanone intake. These findings are partially in line with those related to flavonoid intake found in the present study, including, but not limited to, the association between flavonol consumption and lower likelihood of having depressive symptoms. Evidence from intervention studies showed, in general, significant results in improving mood or ameliorating symptoms of depression. Results from the present study are in line with intervention trials showing potential effects of cocoa- [31], berry- [32], citrus- [33], legume- and wine- [34], and tea-polyphenols [35] against depressive symptoms. The results from the meta-analysis on the association between dietary components and depression suggest that polyphenol-rich foods may play an important role in preventing depression [7,8]. In this study, we did not find association of individual food groups rich in polyphenols (such as fruit and vegetable) and depressive symptoms, suggesting that the potential beneficial effects are not driven by an individual dietary component but rather its content in specific phenolic compounds. Nonetheless, citrus fruits and red wine consumption was significantly associated with depressive symptoms. Interestingly, the report from NHS and NHSII demonstrated that compared with citrus intake of less than one serving/week, intakes of more than two servings/d were associated with an 18% reduction in depression risk. Moreover, results from other cohorts of adults living in the Mediterranean area showed that moderate alcohol consumption, mainly represented by wine, was associated with lower risk of depression [36,37]. Despite these results must be considered with caution due to the potential detrimental effects of excessive alcohol consumption, further investigations involving wine-polyphenols (i.e., stilbenes and anthocyanins) can be considered in relation to symptoms of depression. Nonetheless, the present study did not found any other association between food groups and depressive symptoms, suggesting that either minimum intake of polyphenols to observe a significant effect is hardly reachable from one unique food source in a normal diet or that a certain degree of synergy between various polyphenol classes and compounds might be needed. Previous reports from this cohort showed that higher intake of flavonoids [23], phenolic acids [38] and phytoestrogens [39] may exert beneficial effects toward health. In contrast, certain compounds (such as lignans, flavonols, and hydroxycinnamic acids) showed null association with depressive symptoms at high intake, while a significant association was found in the third group of exposures. It has been previously suggested that a potentially non-linear association between flavonoid intake and health outcomes may exist [10], despite it is still not clear whether the lack of positive effect depends on flavonoid itself (i.e., high intake of flavonoid may be detrimental through stimulation of pro-oxidant processes) or due to other factors associated with their intake (i.e., high intake of flavonoid may be associated with excess calorie intake or alcohol consumption). This matter is still object of observation and further studies are needed to clarify such issue.

From a mechanistic point of view, growing evidence supports a potential beneficial effect of polyphenols on mood and brain health [40]. It has been suggested that dietary polyphenols may be involved in depression pathophysiology through direct and indirect mechanisms. Direct mechanisms may include suppression of neuronal apoptosis, modulation of signalling pathways implicated in neuron survival, and stimulation of adult neurogenesis [28]. On the other hand, indirect mechanisms may comprise anti-neuroinflammatory properties and reduction of oxidative stress through improved blood flow [28]. Despite the increasing amount of evidence for the bioavailability of polyphenols in the systemic circulation [41], limited information is available regarding their ability to reach the central nervous system (CNS). Studies using in vitro models, demonstrated that polyphenols permeation through the blood-brain barrier (BBB) depends on the degree of lipophilicity of the compound, with less polar polyphenols or metabolites capable of greater brain uptake than the more polar ones [42]. For example, naringenin was found in brain tissue after intravenous administration [43], while anthocyanins after oral administration [44,45]. Finally, the data suggest that the concentration of polyphenols in brain tissue may reach approximately 1 nmol/g tissue [46], in different brain regions, and generally in non-specific manner [44,45]. Another intriguing indirect mechanism has been suggested to be the influence of polyphenols on gut microbiota. Indeed, there is evidence that the gut microbiota may affect brain and behaviors of relevance to anxiety and that its manipulation may influence depression-like behaviors [47]. The mechanisms rely on the modification of bacterial ratios through difference polyphenol classes intake determining pro and anti-inflammatory balance in the gut, which in turn may have an impact at systemic level. The literature from this field is just emerging and there is a need for a critical mass of high-quality research to investigate the potential association between polyphenol intake, gut microbiota and mental health.

With a special regard to the individual subclasses of polyphenols demonstrated to be associated with depressive symptoms in the present study, current scientific evidence from animal models suggest the potential involvement of anthocyanins in inhibiting monoamine oxidases (MAOs), mitochondrial enzymes that catalyse the oxidation of monoamines, which elevated activity has been associated with depression [48]. Hydroxycinnamic acids were shown to modulate the parameters of neuro-inflammation [49]. Moreover flavanones (i.e., hesperetin) were shown to exert antidepressant-like activity dependent on interaction with the serotoninergic and kappa opioidergic receptors [50]. Nevertheless, there are some questionable issues to be addressed. For instance, polyphenol absorption, metabolism, and disposition in tissues and cells highly depends on the chemical structure of the compounds, with a wide range of variability between and within classes [41]. Moreover, in order to exert the aforementioned effects in human brain, polyphenols must cross the BBB at pharmacologically effective concentrations [51].

The present study has the strength to investigate the association between a wide range of polyphenol classes, subclasses and individual compounds, polyphenol-rich foods and depressive symptoms for the first time. However, some limitations should be pointed out before considering the results. First, the cross-sectional nature of the study does not allow defining causal relation; despite we may hypothesize that dietary habits are relatively stable among the individuals recruited for the study, we cannot rule out the possibility of reverse causation. Second, a common limitation of studies in nutritional epidemiology assessing food intake rather than biomarkers is the possibility of collinearity, as many phytochemicals are contained in the same food sources. Thus, it is not possible to disentangle the real association of an individual compound from the others. Third, the use of FFQs may be subject to recall bias and potential inaccuracy in estimating micronutrients or other compounds. Finally, considering the stability of the results after adjustment for a wide range of lifestyle and dietary confounding factors, including vitamins and adherence to the Mediterranean diet, a residual confounding is unlikely.

## 4. Materials and Methods

### 4.1. Study Design and Population

The MEAL study is an observational study designed in order to investigate the relationship between nutritional and lifestyle habits characterizing the classical Mediterranean area and non-communicable diseases. The baseline data included a sample of 2044 men and women aged 18 or more years old, randomly selected in the main districts of the city of Catania, southern Italy. Details of the study protocol are published elsewhere [52]. Briefly, the enrolment and data collection was performed between 2014 and 2015 by extracting potential participants from the list of a pool of general practitioners’ databases. A theoretical sample size was hypothesized to include 1500 individuals (providing a specific relative precision of 5%—Type I error, 0.05; Type II error, 0.10—and an anticipated 70% participation rate). Potential participants were stratified by age groups of 10 years and sex. Pregnant women and patients with physical or mental impossibility to participate to the study were not considered for the selection. Out of 2405 individuals invited, the final sample size was 2044 participants (response rate of 85%). All participants were informed about the aims of the study and provided a written informed consent. All the study procedures were carried out in accordance with the Declaration of Helsinki (1989) of the World Medical Association. The study protocol has been reviewed and approved by the concerning ethical committee (protocol number: 802/23 December 2014).

### 4.2. Data Collection

An electronic data collection was performed by face-to-face assisted personal interview, using tablet computers. In order to visualize the response options, participants were provided with a paper copy of the questionnaire. However, final answers were registered directly by the interviewer. The demographic data included gender, age at recruitment, highest educational degree achieved, occupation (specifies the character of the most important employment during the year before the investigation) or last occupation before retirement, and marital status. Educational status was categorized as: (i) low (primary/secondary), (ii) medium (high school), and (iii) high (university). Occupational status was categorized as: (i) unemployed, (ii) low (unskilled workers), (iii) medium (partially skilled workers), and (iv) high (skilled workers). Physical activity status was evaluated using International Physical Activity Questionnaires (IPAQ) [53], which included a set of questionnaires (five domains) investigating the time spent being physically active in the last seven days. Based on the IPAQ guidelines, final score allows categorizing physical activity level as (i) low, (ii) moderate, and (iii) high. Smoking status was categorized as: (i) non-smoker, (ii) ex-smoker, and (iii) current smoker. Alcohol consumption was categorized as: (i) none, (ii) moderate drinker (0.1–12 g/d) and (iii) regular drinker (>12 g/d).

### 4.3. Health Status

Anthropometric measurements have been collected following standard procedures [54]. Height was measured to the nearest 0.5 cm without shoes, with the back square against the wall tape, eyes looking straight ahead, with a right-angle triangle resting on the scalp and against the wall. Weight was measured with a lever balance to the nearest 100 g without shoes and with light undergarments. Body mass index (BMI) was calculated, and patients were categorized as under/normal weight (BMI < 25 Kg/m^2^), overweight (BMI 25 to 29.9 Kg/m^2^), and obese (BMI > 30 Kg/m^2^). Arterial blood pressure was measured in sitting position and at least after 5 minutes of rest, at the end of the physical examination. Because of the possibility of differences in blood pressure measurement, the measurements were taken three times at the right arm relaxed and well supported by a table, with an angle of 45° from the trunk. A mean of the last two measurements was considered for inclusion in the database. Patients have been considered hypertensive when average systolic/diastolic blood pressure levels were higher or equal to 140/90 mm Hg, taking anti-hypertensive medications, or being previously diagnosed of hypertension. Database records were checked to assess diagnosis of type 2 diabetes, dyslipidemias, previous episodes of cardiovascular disease or cancer. Data on prescription of antidepressants were also collected, but no participant was previously administered with such drugs.

### 4.4. Dietary Assessment

The dietary assessment has been performed by the administration of two food frequency questionnaires (FFQ, a long and a short version) that have been previously tested for validity and reliability for the Sicilian population [55,56]. The identification of the food intake, the energy content as well as the macro- and micro-nutrients intake were obtained through comparison with food composition tables of the Research Center for Foods and Nutrition [57]. Intake of seasonal foods referred to consumption during the period in which the food was available and then adjusted by its proportional intake in one year. FFQs with unreliable intakes (<1000 or >6000 kcal/d) were excluded from the analyses (*n* = 107) leaving a total of 1937 individuals for further consideration for the analysis.

The Mediterranean diet adherence was assessed using a standardized score (MEDI-LITE score) [58]: briefly, a scoring system was built weighting all the median (or mean) values for the sample size of each study population and then calculating a mean value of all the weighted medians. Hence, two standard deviations were used to determine three different categories of consumption for each food group. For food groups, typical to the Mediterranean diet (fruit, vegetables, cereals, legumes and fish), 2 points were given for the highest category of consumption, 1 point for the middle category and 0 point for the lowest category. Conversely, for food groups not typical to the Mediterranean diet (meat and meat products, dairy products), 2 points were given for the lowest category, 1 point for the middle category and 0 point for the highest category of consumption. For alcohol, categories related to the alcohol unit (1 alcohol unit = 12 g of alcohol) were used by giving 2 points for the middle category (1–2 alcohol units/d), 1 point for the lowest category (<1 alcohol unit/d) and 0 point for the highest category of consumption (>2 alcohol units/d). The final adherence score comprised nine food categories (including olive oil) with a score ranging from 0 point (lowest adherence) to 18 points (highest adherence).

### 4.5. Estimation of Polyphenol Intake

The process of the estimation of habitual polyphenols intakes has been previously described in detail [59]. Briefly, data on the polyphenol content in foods were retrieved from the Phenol-Explorer database (www.phenol-explorer.eu) [60]. A new version of the Phenol-Explorer database containing data on the effects of cooking and food processing on polyphenol contents was used whenever possible in order to apply polyphenol-specific retention factors [61]. Foods that contained no polyphenols were excluded from the calculation, leaving a total of 75 items included in the analyses. Food weight loss or gain during cooking was corrected using yield factors [62]. The average food consumption was calculated (in g or ml) by following the standard portion sizes used in the study, and then converted in 24-h intake. Finally, a search was carried out in the Phenol-Explorer database to retrieve mean content values for all polyphenols contained in the selected foods. Next, polyphenols intake from each food was calculated by multiplying the content of each polyphenol class by the daily consumption of each food. The total polyphenol intake was considered as the sum of all the main classes and subclasses. Finally, polyphenol intake was adjusted for total energy intake (kcal/d) using the residual method [63].

### 4.6. Depressive Symptoms

The 10-item Center for the Epidemiological Studies of Depression Short Form (CES-D-10) was used as screening tool for depressive symptoms [64]. Briefly, CES-D-10 is a 10-item questionnaire widely used to screen for depressive symptoms in general population. CES-D-10 is assessing depressive symptoms in past week; total scores can range from 0 to 30. Higher scores suggest greater severity of symptoms, a score ≥16 indicated was considered as a case with depressive symptoms. After excluding the individuals who did not complete CES-D-10, a total sample of 1572 was included in the final analysis.

### 4.7. Statistical Analysis

Frequencies are expressed as absolute numbers and percentages; continuous variables are expressed as means and standard deviations. Individuals were divided into quartiles of dietary polyphenols intake and distribution of background characteristics were compared between groups. Differences were tested with Chi-square test for categorical variables, ANOVA for continuous variables distributed normally, and Kruskall-Wallis test for variables distributed not normally. Energy-adjusted multivariate logistic regression models were used to test the association between variables of exposure (including total polyphenols, their classes, subclasses and individual compounds) and depressive symptoms occurrence; a multivariate model adjusted for all other background characteristics (body mass index, physical activity, educational status, occupational status, smoking status, alcohol consumption, occurrence of hypertension, diabetes, dyslipidemias, cardiovascular disease, cancer, menopausal status) and dietary factors (intake of vitamin C, vitamin D, vitamin B12, omega-3, and MEDI-LITE score) was also performed to test whether the observed associations were independent from the aforementioned variables. A multivariate regression analysis was also performed to test whether selected food groups (including red wine, beer, coffee, tea, olive oil, fruits, citrus fruits, vegetables, legumes, nuts and seeds) known to be major sources of polyphenols in the present cohort were related to depressive symptoms occurrence [59]. All reported P-values were based on two-sided tests and compared to a significance level of 5%. SPSS 17 (SPSS Inc., Chicago, IL, USA) software was used for all the statistical calculations.

## 5. Conclusions

In conclusion, selected classes of polyphenols and individual compounds may be associated with aspects of depression. However, further research is needed in order to better understand dose ranges needed to exert protective effects toward depression pathophysiology, and to corroborate whether the associations observed are secondary to a direct (i.e., action on inflammatory pathways in depressive disorders) or indirect (diet quality leading to better general health) impact on markers of mental health.

## Figures and Tables

**Table 1:** Background characteristics of participants in the MEAL cohort by quartiles of total polyphenol intake (energy-adjusted).

	Total Polyphenol Quartiles				*p*
	Q1	Q2	Q3	Q4	
Age (years), mean (SD)	45.29 (19.09)	46.87 (17.66)	47.90 (16.21)	46.30 (15.87)	0.203
Men, *n* (%)	158 (43.1)	186 (45.7)	156 (39.9)	160 (39.3)	0.120
BMI, mean (SD)	25.75 (4.41)	25.64 (4.58)	25.95 (4.45)	25.23 (4.38)	0.167
Smoking status, *n* (%)					0.423
Current	40 (10.9)	47 (11.5)	42 (10.5)	49 (12.3)	
Former	75 (20.4)	109 (26.8)	111 (27.7)	89 (22.4)	
Never	252 (68.7)	251 (61.7)	248 (61.8)	259 (65.2)	
Educational level, *n* (%)					0.001
Low	147 (32.7)	185 (37.0)	187 (37.4)	178 (36.6)	
Medium	153 (34.1)	180 (36.0)	213 (42.6)	174 (35.7)	
High	149 (33.2)	135 (27.0)	100 (20.0)	135 (27.7)	
Occupational level, *n* (%)					0.046
Unemployed	90 (23.7)	115 (26.7)	131 (28.8)	125 (31.9)	
Low	64 (16.8)	66 (15.3)	74 (16.3)	62 (15.8)	
Medium	87 (22.9)	126 (29.2)	123 (27.0)	104 (26.5)	
High	139 (36.6)	124 (28.8)	127 (27.9)	101 (25.8)	
Physical activity level, *n* (%)					0.290
Low	74 (20.2)	76 (18.7)	58 (14.5)	69 (17.5)	
Medium	174 (47.4)	215 (52.8)	176 (44.1)	209 (52.9)	
High	119 (32.4)	116 (28.5)	165 (41.4)	117 (29.6)	
Alcohol consumption, *n* (%)					<0.001
None	103 (28.1)	95 (23.3)	52 (13.0)	39 (9.8)	
Moderate (0.1–12 g/d)	257 (70.0)	281 (69.0)	254 (63.3)	213 (53.7)	
Regular (>12 g/d)	7 (1.9)	31 (7.6)	95 (23.7)	145 (36.5)	
Health status, *n* (%)					
Hypertension	193 (52.6)	218 (53.6)	199 (49.6)	155 (39.0)	<0.001
Diabetes	12 (3.3)	32 (7.9)	31 (7.7)	18 (4.5)	0.561
Dyslipidemias	50 (13.6)	63 (15.5)	72 (18.0)	68 (17.1)	0.125
Cardiovascular disease	27 (7.4)	21 (5.2)	32 (8.0)	25 (6.3)	0.978
Cancer	17 (4.6)	14 (3.4)	10 (2.5)	17 (4.3)	0.667
Menopausal status (women only), *n* (%)	142 (54.6)	160 (55.4)	165 (53.1)	166 (55.5)	0.926
MEDI-LITE score, mean (SD)	10.7 (2.4)	12.0 (2.1)	12.8 (2.2)	12.6 (1.9)	<0.001
Total energy intake (kcal/d), mean (SD)	1666.54 (491.14)	1873.39 (533.24)	2050.85 (647.58)	2728.92 (1140.65)	<0.001

**Table 2:** Odds ratios (ORs) and 95% confidence intervals (CIs) for the association between polyphenol intake (total and main classes) and depressive symptoms.

	Polyphenol Quartiles				*p* for Trend
	Q1	Q2	Q3	Q4	
Total polyphenols, mean (SD), mg/d	252.24 (57.49)	432.65 (49.77)	624.24 (82.21)	1321.19 (902.24)	
No. of cases	119	139	121	130	0.766
Model 1 ^a^	1	1.08 (0.80, 1.46)	0.90 (0.66, 1.23)	1.01 (0.72, 1.42)	
Model 2 ^b^	1	1.33 (0.92, 1.92)	1.14 (0.78, 1.68)	1.35 (0.87, 2.09)	
Model 3 ^c^	1	1.51 (1.03, 2.22)	1.37 (0.90, 2.09)	1.59 (0.95, 2.64)	
Total flavonoids, mean (SD), mg/d	86.64 (25.08)	157.07 (23.00)	256.52 (37.98)	543.71 (219.55)	
No. of cases	152	103	111	143	0.293
Model 1 ^a^	1	0.52 (0.38, 0.71)	0.59 (0.43, 0.80)	0.82 (0.60, 1.12)	
Model 2 ^b^	1	0.46 (0.32, 0.68)	0.44 (0.30, 0.65)	0.75 (0.50, 1.12)	
Model 3 ^c^	1	0.48 (0.33, 0.71)	0.49 (0.32, 0.75)	0.86 (0.51, 1.43)	
Phenolic acids, mean (SD), mg/d	114.68 (30.98)	203.99 (25.79)	313.22 (40.39)	848.18 (987.59)	
No. of cases	138	102	132	117	0.423
Model 1 ^a^	1	0.65 (0.48, 0.89)	0.97 (0.72, 1.31)	0.77 (0.57, 1.05)	
Model 2 ^b^	1	0.61 (0.42, 0.88)	0.63 (0.44, 0.91)	0.60 (0.41, 0.88)	
Model 3 ^c^	1	0.64 (0.44, 0.92)	0.66 (0.45, 0.95)	0.64 (0.44, 0.93)	
Stilbenes, mean (SD), mg/d	0.06 (0.03)	0.24 (0.08)	0.92 (0.41)	5.66 (4.39)	
No. of cases	119	139	129	122	0.385
Model 1 ^a^	1	1.06 (0.78, 1.43)	0.87 (0.64, 1.19)	0.92 (0.67,1.26)	
Model 2 ^b^	1	1.05 (0.70, 1.57)	0.62 (0.40, 0.98)	0.75 (0.44, 1.26)	
Model 3^c^	1	1.13 (0.75, 1.71)	0.69 (0.44, 1.11)	0.81 (0.47, 1.41)	
Lignans, mean (SD), mg/d	0.73 (0.26)	1.45 (0.26)	2.59 (0.51)	6.37 (3.51)	
No. of cases	159	118	120	112	<0.001
Model 1 ^a^	1	0.52 (0.39, 0.71)	0.49 (0.36, 0.67)	0.55 (0.40, 0.75)	
Model 2 ^b^	1	0.54 (0.37, 0.79)	0.57 (0.39, 0.83)	0.69 (0.47, 1.03)	
Model 3 ^c^	1	0.62 (0.42, 0.91)	0.73 (0.49, 1.09)	1.21 (0.72, 2.04)	

Model 1 ^a^ Adjusted for total energy intake (continuous). Model 2 ^b^ Adjusted as in Model 1, and age, sex, body mass index (continuous), physical activity (low/medium/high), educational status (low/medium/high), occupational status (unemployed/low/medium/high), smoking status (current/former/never), alcohol consumption (no/moderate/regular), occurrence of hypertension, diabetes, dyslipidemias, cardiovascular disease, cancer (yes/no), menopausal status (women only, yes/no). Model 3 ^c^ Adjusted as in Model 2, and intake of vitamin C, vitamin D, vitamin B12, folate, omega-3, and MEDI-LITE score.

**Table 3:** Odds ratios (ORs) and 95% confidence intervals (CIs) for the association between flavonoid and phenolic acid subclasses and depressive symptoms.

	Polyphenol Quartiles				*p* for Trend
	Q1	Q2	Q3	Q4	
Flavonoids					
Flavanols, mean (SD), mg/d	51.45 (40.19)	200.15 (25.43)	292.63 (35.87)	587.05 (179.23)	
No. of cases					<0.001
Model 1 ^a^	1	0.69 (0.51, 0.94)	1.32 (0.98, 1.78)	1.12 (0.82, 1.52)	
Model 2 ^b^	1	0.66 (0.45, 0.96)	1.23 (0.84, 1.80)	1.17 (0.79, 1.73)	
Model 3 ^c^	1	0.71 (0.48, 1.04)	1.35 (0.91, 2.01)	1.25 (0.81, 1.91)	
Flavonols, mean (SD), mg/d	17.23 (6.13)	34.06 (4.29)	55.85 (10.54)	120.76 (49.12)	
No. of cases	144	125	103	137	0.034
Model 1 ^a^	1	0.69 (0.51, 0.93)	0.54 (0.39, 0.73)	0.75 (0.55, 1.02)	
Model 2 ^b^	1	0.65 (0.45, 0.95)	0.53 (0.36, 0.77)	0.78 (0.54, 1.13)	
Model 3 ^c^	1	0.64 (0.43, 0.93)	0.51 (0.34, 0.77)	0.67 (0.42, 1.06)	
Flavanones, mean (SD), mg/d	5.46 (3.20)	16.49 (3.65)	34.32 (8.81)	96.79 (44.95)	
No. of cases	129	157	124	99	<0.001
Model 1 ^a^	1	0.94 (0.70, 1.27)	0.70 (0.51, 0.96)	0.48 (0.34, 0.66)	
Model 2 ^b^	1	0.95 (0.66, 1.37)	0.67 (0.46, 0.98)	0.51 (0.34, 0.75)	
Model 3 ^c^	1	1.00 (0.69, 1.46)	0.67 (0.45, 0.99)	0.54 (0.32, 0.91)	
Flavones, mean (SD), mg/d	2.15 (0.77)	4.37 (0.69)	7.47 (1.45)	19.99 (16.09)	
No. of cases	122	138	126	123	0.022
Model 1 ^a^	1	0.87 (0.64, 1.18)	0.76 (0.55, 1.04)	0.68 (0.49, 0.94)	
Model 2 ^b^	1	0.92 (0.63, 1.34)	1.10 (0.76, 1.60)	0.85 (0.58, 1.25)	
Model 3 ^c^	1	0.99 (0.68, 1.46)	1.38 (0.93, 2.05)	1.04 (0.68, 1.61)	
Anthocyanins, mean (SD), mg/d	11.48 (4.79)	27.28 (5.12)	50. 21 (10.15)	132.29 (67.36)	
No. of cases	138	138	120	113	0.001
Model 1 ^a^	1	0.73 (0.54, 0.98)	0.61 (0.45, 0.83)	0.58 (0.42, 0.81)	
Model 2 ^b^	1	0.82 (0.56, 1.20)	0.57 (0.39, 0.85)	0.57 (0.37, 0.87)	
Model 3 ^c^	1	0.90 (0.61, 1.33)	0.64 (0.43, 0.94)	0.61 (0.42, 0.89)	
Isoflavones, mean (SD), mg/d	0.01 (0.01)	0.03 (0.01)	0.07 (0.01)	17.65 (26.76)	
No. of cases	130	125	107	147	0.347
Model 1 ^a^	1	0.76 (0.56, 1.03)	0.55 (0.40, 0.75)	0.94 (0.69, 1.28)	
Model 2 ^b^	1	0.70 (0.48, 1.01)	0.76 (0.53, 1.09)	0.77 (0.53, 1.12)	
Model 3 ^c^	1	0.73 (0.51, 1.06)	0.84 (0.58, 1.23)	0.73 (0.48, 1.09)	
Phenolic acids					
Hydroxybenzoic acids, mean (SD), mg/d	13.08 (11.51)	64.21 (6.87)	136.26 (35.37)	638.70 (971.59)	
No. of cases	124	126	139	120	0.637
Model 1 ^a^	1	0.78 (0.57, 1.05)	0.96 (0.71, 1.30)	0.85 (0.62, 1.17)	
Model 2 ^b^	1	0.70 (0.49, 1.00)	1.25 (0.86, 1.83)	0.99 (0.67, 1.46)	
Model 3 ^c^	1	0.72 (0.50, 1.02)	1.23 (0.83, 1.82)	0.91 (0.61, 1.37)	
Hydroxycinnamic acids, mean (SD), mg/d	63.99 (15.05)	106.59 (13.02)	156.86 (17.61)	277.67 (96.17)	
No. of cases	144	148	96	121	0.007
Model 1 ^a^	1	0.85 (0.64, 1.14)	0.52 (0.38, 0.71)	0.72 (0.52, 1.00)	
Model 2 ^b^	1	0.87 (0.60, 1.25)	0.59 (0.40, 0.86)	0.81 (0.54, 1.20)	
Model 3 ^c^	1	0.91 (0.63, 1.32)	0.65 (0.44, 0.97)	0.88 (0.57, 1.37)	

Model 1 ^a^ Adjusted for total energy intake (continuous). Model 2 ^b^ Adjusted as in Model 1, and age, sex, body mass index (continuous), physical activity (low/medium/high), educational status (low/medium/high), occupational status (unemployed/low/medium/high), smoking status (current/former/never), alcohol consumption (no/moderate/regular), occurrence of hypertension, diabetes, dyslipidemias, cardiovascular disease, cancer (yes/no), menopausal status (women only, yes/no). Model 3 ^c^ Adjusted as in Model 2, and intake of vitamin C, vitamin D, vitamin B12, folate, omega-3, and MEDI-LITE score.

**Table 4:** Odds ratios (ORs) and 95% confidence intervals (CIs) for the association between individual phenolic compounds and depressive symptoms.

	Polyphenol Quartiles			
	Q1	Q2	Q3	Q4
Flavonoids				
Flavonols				
Quercetin				
Model 1 ^a^	1	0.60 (0.40, 0.89)	0.91 (0.62, 1.33)	0.48 (0.31, 0.75)
Model 2 ^b^	1	0.60 (0.40, 0.91)	0.98 (0.66, 1.45)	0.53 (0.32, 0.86)
Myricetin				
Model 1 ^a^	1	0.79 (0.28, 2.22)	0.41 (0.14, 1.17)	0.93 (0.32, 2.69)
Model 2 ^b^	1	0.83 (0.29, 2.35)	0.44 (0.15, 1.25)	0.98 (0.33, 2.87)
Kaempferol				
Model 1 ^a^	1	0.69 (0.47, 1.01)	0.56 (0.39, 0.82)	0.55 (0.34, 0.88)
Model 2 ^b^	1	0.74 (0.49, 1.09)	0.63 (0.41, 0.97)	0.66 (0.38, 1.13)
Flavanols				
Catechins				
Model 1 ^a^	1	0.84 (0.62, 1.13)	1.28 (0.96, 1.70)	1.30 (0.97, 1.76)
Model 2 ^b^	1	0.90 (0.62, 1.31)	1.24 (0.84, 1.83)	1.55 (0.96, 2.50)
Flavanones				
Hesperetin				
Model 1 ^a^	1	1.00 (0.70, 1.45)	0.66 (0.45, 0.96)	0.56 (0.38, 0.82)
Model 2 ^b^	1	1.07 (0.74, 1.55)	0.67 (0.45, 0.99)	0.64 (0.38, 1.09)
Naringenin				
Model 1 ^a^	1	0.49 (0.34, 0.72)	0.30 (0.20, 0.45)	0.42 (0.28, 0.63)
Model 2 ^b^	1	0.50 (0.34, 0.73)	0.31 (0.20, 0.47)	0.51 (0.30, 0.85)
Flavones				
Apigenin				
Model 1 ^a^	1	1.09 (0.73, 1.62)	1.24 (0.81, 1.89)	0.94 (0.63, 1.39)
Model 2 ^b^	1	1.15 (0.76, 1.72)	1.29 (0.83, 1.99)	1.09 (0.72, 1.66)
Luteolin				
Model 1 ^a^	1	0.59 (0.40, 0.86)	0.94 (0.65, 1.35)	0.87 (0.59, 1.29)
Model 2 ^b^	1	0.62 (0.42, 0.91)	1.05 (0.72, 1.53)	1.02 (0.68, 1.55)
Isoflavones				
Daidzein				
Model 1^a^	1	0.77 (0.53, 1.11)	0.83 (0.57, 1.19)	0.85 (0.58, 1.25)
Model 2 ^b^	1	0.82 (0.56, 1.19)	0.92 (0.63, 1.34)	0.84 (0.55, 1.27)
Genistein				
Model 1 ^a^	1	0.81 (0.56, 1.16)	0.79 (0.55, 1.16)	0.78 (0.53, 1.14)
Model 2 ^b^	1	0.86 (0.59, 1.23)	0.88 (0.59, 1.31)	0.75 (0.49, 1.13)
Biochanin A				
Model 1 ^a^	1	1.48 (0.98, 2.25)	1.24 (0.81, 1.89)	1.57 (0.98, 2.47)
Model 2 ^b^	1	1.57 (1.03, 2.38)	1.29 (0.84, 1.98)	1.56 (0.99, 2.42)
Phenolic acids				
Hydroxycinnamic acids				
Caffeic acid				
Model 1 ^a^	1	0.78 (0.53, 1.14)	0.72 (0.48, 1.06)	1.00 (0.59, 1.69)
Model 2 ^b^	1	0.80 (0.54, 1.19)	0.81 (0.53, 1.24)	1.16 (0.67, 2.02)
Cinnamic acid				
Model 1 ^a^	1	0.84 (0.58, 1.21)	1.30 (0.88, 1.92)	1.24 (0.85, 1.80)
Model 2 ^b^	1	0.88 (0.61, 1.28)	1.47 (0.99, 2.19)	1.35 (0.92, 1.99)
Ferulic acid				
Model 1 ^a^	1	0.93 (0.64, 1.35)	1.09 (0.75, 1.57)	1.18 (0.78, 1.78)
Model 2 ^b^	1	1.03 (0.69, 1.51)	1.19 (0.82, 1.75)	1.45 (0.93, 2.25)
Hydroxybenzoic acids				
Vanillic acid				
Model 1 ^a^	1	0.44 (0.29, 0.66)	0.86 (0.58, 1.26)	0.83 (0.54, 1.28)
Model 2 ^b^	1	0.44 (0.29, 0.67)	0.88 (0.59, 1.31)	0.89 (0.56, 1.39)
Lignans				
Lariciresinol				
Model 1 ^a^	1	0.51 (0.35, 0.74)	0.42 (0.29, 0.61)	0.62 (0.42, 0.91)
Model 2 ^b^	1	0.57 (0.39, 0.83)	0.51 (0.34, 0.76)	0.96 (0.58, 1.58)
Matairesinol				
Model 1^a^	1	0.60 (0.41, 0.86)	0.30 (0.20, 0.44)	0.49 (0.33, 0.74)
Model 2 ^b^	1	0.63 (0.43, 0.91)	0.32 (0.21, 0.49)	0.62 (0.37, 1.04)
Pinoresinol				
Model 1 ^a^	1	0.56 (0.38, 0.82)	0.77 (0.53, 1.12)	0.79 (0.53, 1.17)
Model 2 ^b^	1	0.64 (0.44, 0.95)	1.03 (0.69, 1.56)	1.55 (0.92, 2.62)
Secoisolariciresinol				
Model 1 ^a^	1	0.74 (0.51, 1.08)	0.73 (0.49, 1.08)	1.05 (0.70, 1.57)
Model 2 ^b^	1	0.82 (0.56, 1.20)	1.08 (0.70, 1.66)	2.21 (0.97, 4.07)

Model 1 ^a^ Adjusted for total energy intake (continuous). Model 2 ^b^ Adjusted as in Model 1, and age, sex, body mass index (continuous), physical activity (low/medium/high), educational status (low/medium/high), occupational status (unemployed/low/medium/high), smoking status (current/former/never), alcohol consumption (no/moderate/regular), occurrence of hypertension, diabetes, dyslipidemias, cardiovascular disease, cancer (yes/no), menopausal status (women only, yes/no), intake of vitamin C, vitamin D, vitamin B12, folate, omega-3, and MEDI-LITE score.

**Table 5:** Odds ratios (ORs)^a^ and 95% confidence intervals (CIs) for the association between selected major food sources of polyphenols and depressive symptoms.

	Food Quartiles, OR (95% CI)^a^		
	Q1	Q2	Q3	Q4
Red wine	1	1.12 (0.77, 1.62)	0.22 (0.08, 0.60)	0.53 (0.38, 0.74)
Beer	1	0.78 (0.53, 1.14)	0.81 (0.51, 1.29)	0.60 (0.26, 1.38)
Coffee	1	2.10 (1.14, 3.89)	1.66 (0.94, 2.90)	1.41 (0.81, 2.44)
Tea	1	0.97 (0.73, 1.27)	1.03 (0.65, 1.64)	1.09 (0.33, 3.52)
Olive oil	1	2.35 (0.59, 9.61)	1.48 (0.37, 5.81)	1.97 (0.50, 7.70)
Fruits	1	0.79 (0.55, 1.15)	0.57 (0.39, 0.83)	0.88 (0.60, 1.30)
Citrus fruits	1	0.58 (0.41, 0.83)	0.36 (0.25, 0.52)	0.51 (0.35, 0.75)
Vegetables	1	1.08 (0.75, 1.56)	0.98 (0.67, 1.42)	1.23 (0.83, 1.82)
Legumes	1	0.94 (0.66, 1.33)	1.00 (0.69, 1.45)	0.67 (0.46, 1.00)
Nuts and seeds	1	0.89 (0.65, 1.22)	0.67 (0.41, 1.10)	0.80 (0.45, 1.41)

^a^ Adjusted for age (continuous), sex, total energy intake (continuous), body mass index (continuous), physical activity (low/medium/high), educational status (low/medium/high), occupational status (unemployed/low/ medium/high), smoking status (current/former/never), alcohol consumption (no/moderate/regular), occurrence of hypertension, diabetes, dyslipidemias, cardiovascular disease, cancer (yes/no), menopausal status (women only, yes/no).

## Supplementary Materials

The following are available online at http://www.mdpi.com/1420-3049/23/5/999/s1, Table S1: Selected dietary factors by quartiles of total polyphenol intake (energy-adjusted).

## References

[1] Disease G.B.D., Injury I., Prevalence C. (2016). Global, regional, and national incidence, prevalence, and years lived with disability for 310 diseases and injuries, 1990–2015: A systematic analysis for the global burden of disease study 2015. Lancet.

[2] Olesen J., Gustavsson A., Svensson M., Wittchen H.U., Jonsson B., The CDBE2010 study group, The European Brain Council (2012). The economic cost of brain disorders in Europe. Eur. J. Neurol..

[3] Grosso G., Galvano F., Marventano S., Malaguarnera M., Bucolo C., Drago F., Caraci F. (2014). Omega-3 fatty acids and depression: Scientific evidence and biological mechanisms. Oxid. Med. Cell. Longev..

[4] Quirk S.E., Williams L.J., O’Neil A., Pasco J.A., Jacka F.N., Housden S., Berk M., Brennan S.L. (2013). The association between diet quality, dietary patterns and depression in adults: A systematic review. BMC Psychiatry.

[5] Molendijk M., Molero P., Ortuno Sanchez-Pedreno F., Van der Does W., Angel Martinez-Gonzalez M. (2018). Diet quality and depression risk: A systematic review and dose-response meta-analysis of prospective studies. J. Affect. Disord..

[6] Li Y., Lv M.R., Wei Y.J., Sun L., Zhang J.X., Zhang H.G., Li B. (2017). Dietary patterns and depression risk: A meta-analysis. Psychiatry Res..

[7] Grosso G., Micek A., Castellano S., Pajak A., Galvano F. (2016). Coffee, tea, caffeine and risk of depression: A systematic review and dose-response meta-analysis of observational studies. Mol. Nutr. Food Res..

[8] Liu X., Yan Y., Li F., Zhang D. (2016). Fruit and vegetable consumption and the risk of depression: A meta-analysis. Nutrition.

[9] Grosso G., Godos J., Lamuela-Raventos R., Ray S., Micek A., Pajak A., Sciacca S., D’Orazio N., Del Rio D., Galvano F. (2017). A comprehensive meta-analysis on dietary flavonoid and lignan intake and cancer risk: Level of evidence and limitations. Mol. Nutr. Food Res..

[10] Grosso G., Micek A., Godos J., Pajak A., Sciacca S., Galvano F., Giovannucci E.L. (2017). Dietary flavonoid and lignan intake and mortality in prospective cohort studies: Systematic review and dose-response meta-analysis. Am. J. Epidemiol..

[11] Tang Z., Li M., Zhang X., Hou W. (2016). Dietary flavonoid intake and the risk of stroke: A dose-response meta-analysis of prospective cohort studies. BMJ Open.

[12] Wang X., Ouyang Y.Y., Liu J., Zhao G. (2014). Flavonoid intake and risk of CVD: A systematic review and meta-analysis of prospective cohort studies. Br. J. Nutr..

[13] Godos J., Rapisarda G., Marventano S., Galvano F., Mistretta A., Grosso G. (2017). Association between polyphenol intake and adherence to the Mediterranean diet in Sicily, southern Italy. NFS J..

[14] Mocciaro G., Ziauddeen N., Godos J., Marranzano M., Chan M.Y., Ray S. (2017). Does a mediterranean-type dietary pattern exert a cardio-protective effect outside the Mediterranean region? A review of current evidence. Int. J. Food Sci. Nutr..

[15] Platania A., Zappala G., Mirabella M.U., Gullo C., Mellini G., Beneventano G., Maugeri G., Marranzano M. (2017). Association between Mediterranean diet adherence and dyslipidaemia in a cohort of adults living in the Mediterranean area. Int. J. Food Sci. Nutr..

[16] Beunza J.J., Toledo E., Hu F.B., Bes-Rastrollo M., Serrano-Martinez M., Sanchez-Villegas A., Martinez J.A., Martinez-Gonzalez M.A. (2010). Adherence to the mediterranean diet, long-term weight change, and incident overweight or obesity: The seguimiento universidad de navarra (sun) cohort. Am. J. Clin. Nutr..

[17] Martinez-Gonzalez M.A., Garcia-Arellano A., Toledo E., Salas-Salvado J., Buil-Cosiales P., Corella D., Covas M.I., Schroder H., Aros F., Gomez-Gracia E. (2012). A 14-item mediterranean diet assessment tool and obesity indexes among high-risk subjects: The predimed trial. PLoS ONE.

[18] Mendez M.A., Popkin B.M., Jakszyn P., Berenguer A., Tormo M.J., Sanchez M.J., Quiros J.R., Pera G., Navarro C., Martinez C. (2006). Adherence to a mediterranean diet is associated with reduced 3-year incidence of obesity. J. Nutr..

[19] Mistretta A., Marventano S., Antoci M., Cagnetti A., Giogianni G., Nolfo F., Rametta S., Pecora G., Marranzano M. (2017). Mediterranean diet adherence and body composition among southern Italian adolescents. Obes. Res. Clin. Pract..

[20] Zappala G., Buscemi S., Mule S., La Verde M., D’Urso M., Corleo D., Marranzano M. (2017). High adherence to Mediterranean diet, but not individual foods or nutrients, is associated with lower likelihood of being obese in a Mediterranean cohort. Eat. Weight Disord..

[21] La Verde M., Mule S., Zappala G., Privitera G., Maugeri G., Pecora F., Marranzano M. (2018). Higher adherence to the Mediterranean diet is inversely associated with having hypertension: Is low salt intake a mediating factor?. Int. J. Food Sci. Nutr..

[22] Nunez-Cordoba J.M., Valencia-Serrano F., Toledo E., Alonso A., Martinez-Gonzalez M.A. (2009). The mediterranean diet and incidence of hypertension: The Seguimiento Universidad de Navarra (SUN) Study. Am. J. Epidemiol..

[23] Marranzano M., Sumantra R., Godos J., Galvano F. (2018). Association between dietary flavonoids intake and obesity in a cohort of adults living in the Mediterranean area. Int. J. Food Sci. Nutr..

[24] Muros J.J., Cofre-Bolados C., Arriscado D., Zurita F., Knox E. (2017). Mediterranean diet adherence is associated with lifestyle, physical fitness, and mental wellness among 10-y-olds in Chile. Nutrition.

[25] Bonaccio M., Di Castelnuovo A., Costanzo S., Pounis G., Persichillo M., Cerletti C., Donati M.B., de Gaetano G., Iacoviello L. (2018). Mediterranean-type diet is associated with higher psychological resilience in a general adult population: Findings from the Moli-sani study. Eur. J. Clin. Nutr..

[26] Sanchez-Villegas A., Martinez-Gonzalez M.A., Estruch R., Salas-Salvado J., Corella D., Covas M.I., Aros F., Romaguera D., Gomez-Gracia E., Lapetra J. (2013). Mediterranean dietary pattern and depression: The predimed randomized trial. BMC Med..

[27] Sureda A., Tejada S. (2015). Polyphenols and depression: From chemistry to medicine. Curr. Pharm. Biotechnol..

[28] Rendeiro C., Rhodes J.S., Spencer J.P. (2015). The mechanisms of action of flavonoids in the brain: Direct versus indirect effects. Neurochem. Int..

[29] Chang S.C., Cassidy A., Willett W.C., Rimm E.B., O’Reilly E.J., Okereke O.I. (2016). Dietary flavonoid intake and risk of incident depression in midlife and older women. Am. J. Clin. Nutr..

[30] Wang J., Wu X., Lai W., Long E., Zhang X., Li W., Zhu Y., Chen C., Zhong X., Liu Z. (2017). Prevalence of depression and depressive symptoms among outpatients: A systematic review and meta-analysis. BMJ Open.

[31] Pase M.P., Scholey A.B., Pipingas A., Kras M., Nolidin K., Gibbs A., Wesnes K., Stough C. (2013). Cocoa polyphenols enhance positive mood states but not cognitive performance: A randomized, placebo-controlled trial. J. Psychopharmacol..

[32] Khalid S., Barfoot K.L., May G., Lamport D.J., Reynolds S.A., Williams C.M. (2017). Effects of acute blueberry flavonoids on mood in children and young adults. Nutrients.

[33] Matsumoto T., Asakura H., Hayashi T. (2014). Effects of olfactory stimulation from the fragrance of the Japanese Citrus Fruit Yuzu (citrus junosSieb. Ex Tanaka) on mood states and salivary Chromogranin a as an Endocrinologic stress marker. J. Altern. Complement. Med..

[34] Davinelli S., Scapagnini G., Marzatico F., Nobile V., Ferrara N., Corbi G. (2017). Influence of equol and resveratrol supplementation on health-related quality of life in menopausal women: A randomized, placebo-controlled study. Maturitas.

[35] Rondanelli M., Opizzi A., Solerte S.B., Trotti R., Klersy C., Cazzola R. (2009). Administration of a dietary supplement (N-oleyl-phosphatidylethanolamine and epigallocatechin-3-gallate formula) enhances compliance with diet in healthy overweight subjects: A randomized controlled trial. Br. J. Nutr..

[36] Gea A., Beunza J.J., Estruch R., Sanchez-Villegas A., Salas-Salvado J., Buil-Cosiales P., Gomez-Gracia E., Covas M.I., Corella D., Fiol M. (2013). Alcohol intake, wine consumption and the development of depression: The predimed study. BMC Med..

[37] Gea A., Martinez-Gonzalez M.A., Toledo E., Sanchez-Villegas A., Bes-Rastrollo M., Nunez-Cordoba J.M., Sayon-Orea C., Beunza J.J. (2012). A longitudinal assessment of alcohol intake and incident depression: The sun project. BMC Public Health.

[38] Godos J., Sinatra D., Blanco I., Mule S., La Verde M., Marranzano M. (2017). Association between dietary phenolic acids and hypertension in a Mediterranean cohort. Nutrients.

[39] Godos J., Bergante S., Satriano A., Pluchinotta F.R., Marranzano M. (2018). Dietary phytoestrogen intake is inversely associated with hypertension in a cohort of adults living in the Mediterranean area. Molecules.

[40] Zainuddin M.S., Thuret S. (2012). Nutrition, adult hippocampal neurogenesis and mental health. Br. Med. Bull..

[41] Del Rio D., Rodriguez-Mateos A., Spencer J.P., Tognolini M., Borges G., Crozier A. (2013). Dietary (poly)phenolics in human health: Structures, bioavailability, and evidence of protective effects against chronic diseases. Antioxid. Redox Signal..

[42] Youdim K.A., Qaiser M.Z., Begley D.J., Rice-Evans C.A., Abbott N.J. (2004). Flavonoid permeability across an in situ model of the blood-brain barrier. Free Radic. Biol. Med..

[43] Peng H.W., Cheng F.C., Huang Y.T., Chen C.F., Tsai T.H. (1998). Determination of naringenin and its glucuronide conjugate in rat plasma and brain tissue by high-performance liquid chromatography. J. Chromatogr. B Biomed. Sci. Appl..

[44] Kalt W., Blumberg J.B., McDonald J.E., Vinqvist-Tymchuk M.R., Fillmore S.A., Graf B.A., O’Leary J.M., Milbury P.E. (2008). Identification of anthocyanins in the liver, eye, and brain of blueberry-fed pigs. J. Agric. Food Chem..

[45] Talavera S., Felgines C., Texier O., Besson C., Gil-Izquierdo A., Lamaison J.L., Remesy C. (2005). Anthocyanin metabolism in rats and their distribution to digestive area, kidney, and brain. J. Agric. Food Chem..

[46] Schaffer S., Halliwell B. (2012). Do polyphenols enter the brain and does it matter? Some theoretical and practical considerations. Genes Nutr..

[47] Dash S., Clarke G., Berk M., Jacka F.N. (2015). The gut microbiome and diet in psychiatry: Focus on depression. Curr. Opin. Psychiatry.

[48] Grosso C., Valentao P., Ferreres F., Andrade P.B. (2013). The use of flavonoids in central nervous system disorders. Curr. Med. Chem..

[49] Hall S., Desbrow B., Anoopkumar-Dukie S., Davey A.K., Arora D., McDermott C., Schubert M.M., Perkins A.V., Kiefel M.J., Grant G.D. (2015). A review of the bioactivity of coffee, caffeine and key coffee constituents on inflammatory responses linked to depression. Food Res. Int..

[50] Pathak L., Agrawal Y., Dhir A. (2013). Natural polyphenols in the management of major depression. Expert Opin. Investig. Drugs.

[51] Andrade P.B., Grosso C., Valentao P., Bernardo J. (2016). Flavonoids in neurodegeneration: Limitations and strategies to cross CNS barriers. Curr. Med. Chem..

[52] Grosso G., Marventano S., D’Urso M., Mistretta A., Galvano F. (2017). The mediterranean healthy eating, ageing, and lifestyle (meal) study: Rationale and study design. Int. J. Food Sci. Nutr..

[53] Craig C.L., Marshall A.L., Sjostrom M., Bauman A.E., Booth M.L., Ainsworth B.E., Pratt M., Ekelund U., Yngve A., Sallis J.F. (2003). International physical activity questionnaire: 12-country reliability and validity. Med. Sci. Sports Exerc..

[54] Mistretta A., Marventano S., Platania A., Godos J., Galvano F., Grosso G. (2017). Metabolic profile of the mediterranean healthy eating, lifestyle and aging (meal) study cohort. Mediterr. J. Nutr. Metab..

[55] Buscemi S., Rosafio G., Vasto S., Massenti F.M., Grosso G., Galvano F., Rini N., Barile A.M., Maniaci V., Cosentino L. (2015). Validation of a food frequency questionnaire for use in Italian adults living in Sicily. Int. J. Food Sci. Nutr..

[56] Marventano S., Mistretta A., Platania A., Galvano F., Grosso G. (2016). Reliability and relative validity of a food frequency questionnaire for Italian adults living in Sicily, southern Italy. Int. J. Food Sci. Nutr..

[57] Istituto Nazionale di Ricerca per gli Alimenti e la Nutrizione (2009). Tabelle di Composizione degli Alimenti.

[58] Sofi F., Dinu M., Pagliai G., Marcucci R., Casini A. (2017). Validation of a literature-based adherence score to Mediterranean diet: The MEDI-LITE score. Int. J. Food Sci. Nutr..

[59] Godos J., Marventano S., Mistretta A., Galvano F., Grosso G. (2017). Dietary sources of polyphenols in the mediterranean healthy eating, aging and lifestyle (meal) study cohort. Int. J. Food Sci. Nutr..

[60] Neveu V., Perez-Jiménez J., Vos F., Crespy V., Du Chaffaut L., Mennen L., Knox C., Eisner R., Cruz J., Wishart D. (2010). Phenol-explorer: An online comprehensive database on polyphenol contents in foods. Database.

[61] Rothwell J.A., Perez-Jimenez J., Neveu V., Medina-Remon A., M’Hiri N., Garcia-Lobato P., Manach C., Knox C., Eisner R., Wishart D.S. (2013). Phenol-explorer 3.0: A major update of the phenol-explorer database to incorporate data on the effects of food processing on polyphenol content. Database.

[62] Bognar A. (2002). Tables on Weight Yield of Food and Retention Factors of Food Constituents for the Calculation of Nutrient Composition of Cooked Foods (Dishes).

[63] Willett W.C.L.E. (1998). Reproducibility and validity of food frequency questionnaire. Nutritional Epidemiology.

[64] Radloff L.S. (1991). The use of the center for epidemiologic studies depression scale in adolescents and young adults. J. Youth Adolesc..

