# Supplementary material for: Dietary Polyphenol Intake and Depression: Results from the Mediterranean Healthy Eating, Lifestyle and Aging (MEAL) Study
Source: Molecules. 2018 Apr 24;23(5):999. doi: 10.3390/molecules23050999 (PMC6102571; doi:10.3390/molecules23050999)
Supplement: Supplementary file 1 [file molecules-23-00999-s001.pdf]

Supplementary Table 1. Background characteristics of participants in the MEAL cohort by presence of depressive symptoms (energy-adjusted).

	Depressive symptoms		
	No (n = 1063)	Yes (n = 509)	*P*
Age (years), mean (SD)	46.9 (16.9)	45.9 (17.9)	0.231
Men, n (%)	473 (44.5)	187 (36.7)	0.004
BMI, mean (SD)	25.7 (4.5)	25.6 (4.4)	0.613
Smoking status, n (%)			0.334
Current	128 (12.0)	50 (9.8)	
Former	252 (23.7)	132 (25.9)	
Never	683 (64.3)	327 (64.2)	
Educational level, n (%)			0.060
Low	300 (28.2)	157 (30.8)	
Medium	457 (43.0)	187 (36.7)	
High	306 (28.8)	165 (32.4)	
Occupational level, n (%)			0.037
Unemployed	217 (24.4)	130 (31.9)	
Low	149 (16.7)	57 (14.0)	
Medium	217 (24.4)	93 (22.9)	
High	307 (34.5)	127 (31.2)	
Physical activity level, n (%)			<0.001
Low	154 (14.5)	123 (24.3)	
Medium	528 (49.8)	246 (48.5)	
High	379 (35.7)	138 (27.2)	
Alcohol consumption, n (%)			0.241
None	207 (19.5)	82 (16.1)	
Moderate (0.1-12 g/d)	674 (63.4)	331 (65.0)	
Regular (>12 g/d)	182 (17.1)	96 (18.9)	
Health status, n (%)			
Hypertension	512 (48.2)	253 (49.7)	0.568
Diabetes	62 (5.8)	31 (6.1)	0.839
Dislipidemia	162 (15.2)	91 (17.9)	0.183
Cardiovascular disease	72 (6.8)	33 (6.5)	0.829
Cancer	38 (3.6)	20 (3.9)	0.727
MEDI-LITE score, mean (SD)	12.2 (2.4)	11.7 (2.2)	<0.001
Menopausal status (women only), n (%)	236 (40)	136 (42.2)	0.511
Total energy intake (kcal/d), mean (SD)	2087.4 (807.7)	2084.5 (932.2)	0.950

Supplementary Table 2. Selected dietary factors by quartiles of total polyphenol intake (energy-adjusted).

	Total polyphenol quartiles			
	Q1	Q2	Q3	Q4
Polyphenols (mg/d), mean (SD)	252.24 (57.49)	432.65 (49.77)	624.24 (82.21)	1321.19 (902.24)
Flavonoids	102.88 (38.61)	183.55 (65.14)	274.38 (96.73)	489.23 (276.30)
Flavonols	29.08 (20.47)	46.85 (29.51)	61.10 (34.22)	94.55 (64.66)
Quercetin	0.25 (0.28)	0.51 (0.56)	0.85 (0.78)	1.33 (1.48)
Myricetin	0.06 (0.13)	0.13 (0.28)	0.35 (0.61)	0.52 (0.83)
Kaempferol	0.09 (0.05)	0.17 (0.11)	0.24 (0.18)	0.39 (0.38)
Flavanols	24.16 (18.69)	54.09 (47.71)	97.27 (73.85)	202.07 (187.54)
Catechins	16.26 (12.99)	36.16 (35.66)	61.73 (53.23)	133.69 (144.24)
Flavanones	18.15 (15.07)	34.06 (30.69)	40.44 (36.97)	64.99 (65.98)
Hesperetin	13.11 (10.89)	24.58 (22.20)	28.96 (26.76)	46.67 (47.41)
Naringenin	2.94 (2.59)	5.38 (5.44)	6.05 (6.15)	10.06 (11.30)
Flavones	4.31 (3.02)	7.21 (5.34)	8.63 (5.30)	15.10 (18.55)
Apigenin	0.007 (0.003)	0.008 (0.003)	0.01 (0.009)	0.009 (0.006)
Luteolin	2.31 (2.08)	3.52 (3.21)	4.20 (3.26)	7.26 (11.93)
Isoflavones	1.36 (5.59)	2.11 (8.30)	4.61 (12.40)	10.12 (25.85)
Daidzein	0.04 (0.11)	0.07 (0.17)	0.13 (0.26)	0.30 (0.75)
Genistein	0.04 (0.13)	0.07 (0.20)	0.13 (0.31)	0.33 (0.89)
Biochanin A	0.0008 (0.0009)	0.001 (0.001)	0.001 (0.001)	0.002 (0.004)
Phenolic acids	129.27 (46.26)	218.94 (70.04)	304.08 (85.07)	781.61 (968.62)
Hydroxycinnamic acids	0.16 (0.16)	0.31 (0.29)	0.41 (0.43)	1.10 (2.22)
Caffeic acid	0.75 (0.43)	1.09 (0.74)	1.85 (1.46)	2.87 (2.25)
Cinnamic acid	0.15 (0.17)	0.29 (0.30)	0.37 (0.50)	1.07 (2.51)
Ferulic acid	1.55 (1.25)	2.20 (1.95)	3.06 (2.53)	5.25 (4.27)
Hydroxybenzoic acids	42.11 (39.86)	96.77 (74.75)	141.35 (94.95)	554.43 (985.37)
Vanillic acid	0.18 (0.18)	0.23 (0.22)	0.43 (0.36)	0.75 (0.74)
Lignans	1.45 (0.95)	2.42 (1.90)	2.82 (2.23)	4.18 (4.03)
Lariciresinol	0.72 (0.55)	1.29 (1.15)	1.52 (1.34)	2.35 (2.45)
Matairesinol	0.01 (0.11)	0.02 (0.02)	0.03 (0.02)	0.05 (0.05)
Pinoresinol	0.53 (0.31)	0.84 (0.58)	0.97 (0.70)	1.39 (1.24)
Secoisolariciresinol	0.06 (0.03)	0.10 (0.07)	0.12 (0.08)	0.17 (0.15)
Stilbenes	0.46 (0.77)	0.88 (1.49)	2.14 (3.33)	3.30 (4.54)
Foods (g/d), mean (SD)				
Red wine	7.70 (16.29)	16.65 (33.96)	42.65 (73.74)	63.53 (100.47)
Beer	30.95 (47.27)	50.39 (96.49)	45.74 (66.72)	95.33 (215.30)
Coffee	42.91 (35.12)	49.83 (39.93)	66.09 (46.38)	61.75 (45.19)
Tea	14.61 (21.59)	41.72 (65.84)	79.00 (103.52)	184.34 (270.45)
Olive oil	6.47 (3.05)	6.79 (2.95)	7.13 (2.91)	6.90 (3.21)
Fruits	246.78 (155.12)	329.03 (226.37)	446.91 (314.95)	648.92 (534.16
Vegetables	169.45 (102.74)	239.76 (97.84)	303.31 (164.63)	392.34 (281.45)
Legumes	23.77 (24.42)	33.18 (27.10)	38.50 (32.36)	64.56 (92.86)
Nuts and seeds	5.90 (4.64)	11.91 (9.86)	15.81 (12.56)	56.24 (85.43)
